# Book Reviews

**Published:** 1816-07

**Authors:** 


### CRITICAL ANALYSIS
### *OF RECENT PUBLICATIONS.*

### Our Reviewer *in Answer to* Dr. Wilson Philip.

*To the Editors of the London Medical and Physical Journal.*

GENTLEMEN,

As you very justly observe, Dr. Wilson Philip having left the public to judge of the matter at issue between us, it was unnecessary to communicate his paper to me before before its publication. On the only remaining difficulty, however, you will allow me to make my defence; after which the decision shall be left to the public, as Dr. W. Philip proposes.

"Hot wires were indeed," says Dr. W. P., "frequently applied to the spinal marrow in my experiments, but this gentleman [meaning his reviewer] conceals the circumstance that it was to the spinal marrow of dead animals *alone* that they were applied."

Now, gentlemen, you and the public shall be left to judge whether, when the experimentor describes certain *actions* consequent upon, or discovered after, the application of hot irons, it is not at least venial for the reader to suppose that the animal was *alive*. I have the honour to be, &c.

---

*Edinburgh Medical and Surgical Journal,* No. XLVI. for April, 1816.

*(Continued from vol. 35, p. 507.)*

Art. V.—*A short Description of an Inflammatory Affection of the Throat (with an Engraving).* By JAMES MURRAY, Surgeon, Belfast.

THIS is a very interesting and important little paper. We could have wished that the engraving had given a better illustration than we found; however the description is, we conceive, quite satisfactory. This we shall transcribe, in order to give the history all the publicity which the author requests, for his own information, and, we add, for the information or minute attention of any other practitioners who may meet with similar cases.

"After exposure to damp and cold, the patients complain of a tickling in the throat, attended with great heat, and the sensation of having been blistered, gradually extending over the palate and Schneiderian membrane, exciting much uneasiness and itching in the nose and all the affected parts.

"On inspecting the throat, the tonsils are found enlarged, and of a dark red colour. The uvula appears soft and papulous, as if composed of a clot of blood. The veins and arteries become distended, and the soft vascular net-work spread over the palate gives it the appearance of a well-injected tissue of blood-vessels.

"The irritability of the surface is such as to cause frequent coughing, and even the least particle of mucus must be thrown up, as if it were some hard substance; blood is also sometimes discharged from the spongy surface of the soft palate and uvula, but without any permanent relief.

"I would anxiously wish for some further elucidation of this subject, whether this obstinate affection has been observed by others, and if any remedy had succeeded in removing it; or whether ther it be supposed a disease *sui generis*, from its resisting all known remedies, or merely a *pseudo-syphilitic* or *mercurial complaint*.

"It is certain that it has generally occurred to those who had used *mercury* for a real or supposed lues; but I have seen three cases where that medicine had never been used, nor any venereal affection even suspected; one of them was a married woman, who had three healthy children during five years that she suffered under it, and had submitted to every plan of cure that could be devised, by numbers of medical gentlemen to whom she had applied. Like all others in this disease, mercury, in any shape or form, was to her invariably injurious.

"In the only case I have seen prove fatal, the uvula and soft palate sloughed away from time to time. Little aphthous crusts formed on the back part of the fauces, falling off successively, and succeeded by round black ulcers, which gradually penetrated deeper and deeper, destroying great part of the nose and internal ears. She could not swallow solid food in this stage of the complaint, her hair fell off, debility and swelling in various parts supervened, and death at length terminated her sufferings.

"In any of the cases I have seen, the constitution exhibited no characteristic mark of a scrofulous diathesis.

"I do not think it necessary to enlarge on this subject at present, nor to enumerate what local and constitutional remedies had been tried, but shall probably, in a short time, submit a few cases of it to your readers, in a detailed manner; and, in the mean time, hope that more light may be thrown on this matter by some abler and more experienced practitioner."

In conformity with the wishes of the author, we shall offer a few conjectures, and such only they must be considered, as we profess never to have seen similar cases pervade a district in the manner here described.

Though exposure to damp and cold are spoken of as the external causes, we take the liberty to hint that these causes are so general as should make us backward in admitting them to be sufficient in themselves.

Enquire then—have the patients slept in an exposed place, with their mouths open, by which means noxious insects may have attached themselves to the diseased parts as a nidus?

Has the disease been confined to a certain district, or does it appear that those who are affected have by any means all breathed the air of any particular spot?

Is it probable that the snuff-box of any individual may have been affected, and others by the common courtesy of offering and accepting the box?

Were those who suffered most great snuff-takers; and were any affected who do not use that herb?

It appears that one woman had three healthy children during her illness: were they subsequently affected by the effluvia from the breath, or in any other way?

Lastly, is a new morbid poison (certainly not sivvins) conveyed by the promiscuous use of the same tobacco-pipe? The succession of sloughs is very similar to the *nigrites serpens* of Celsus, or the *sloughing phagedæna* of some modern writers.

We could wish the engraving had been explained by numerical figures or letters, with references; and should still wish to see the outlines with such references.

We are encouraged to offer these hints by the invitation of Mr. Murray, and, as he is so good as to promise us further information, and more detailed cases, we take the liberty of adding a few more lines.

Only one case, we are told, has proved fatal—Are all the others becoming progressively worse, or are some of them healed spontaneously?

Are the symptoms uniform in all, excepting as to greater or less degree of quickness in their progress?

Have any cases recovered which were exactly similar to the fatal one in its early stage?

On the first appearance of the symptoms, would it be desirable to try the effect of a very strong solution of Kali purum as a gargle, and, if necessary, injected up the nose?

Art. VI.—*Case of Artificial Pupil.* By Mr. MOORE, Surgeon, Belfast.

From what has been written of late years on the subject of artificial pupil, it appears that different methods have been adopted since the original proposal by Mr. Cheselden, and, according to the abettors of each, with greater or less success. The object of the operation being simply that of forming an opening through some part of the iris, as a substitute for the one which has become obliterated by inflammation or accident, or rendered useless by a large central opacity of the cornea, it is manifest that the process must be varied according to the nature and character of the cause which occasioned loss of sight. Hence, under particular circumstances, one mode will be found preferable to another. But, in all, that operation is the most eligible, which, whilst accomplished with least pain and injury to other parts of the organ, leaves the whole in a condition the nearest approaching to its natural state. Every deviation from these particulars must render the eye less sightly and less useful.

From these considerations, and for the reasons stated by Mr. Moore, viz. that "when he has seen it performed, there was a considerable degree of inflammation, and the cure was very tedious," we have always regarded the operation proposed by Sir William Adams as by no means unobjectionable. For, by the method of proceeding which he recommends, not only is great violence done to the organ of vision, and the opening through the iris left of an immoderate size, but we apprehend the danger of subsequent inflammation must also be great, and the absorption of the disorganized lens proportionably slow. By the entire removal of the crystalline, too, even when transparent, the necessity of ever afterwards using convex spectacles becomes indispensable. The plan suggested by our author, if generally practicable, certainly offers several important advantages. He describes it in the following words:

"Having fixed the eye with a speculum, I pierced the cornea with the extracting knife, half a line from the sclerotica on the outside, and a little above the transverse diameter of the cornea, and continued the incision to its lower part, keeping at the same distance from the sclerotica. I then introduced a very small hook, and fixed it in the centre of the iris, carefully avoiding the lens; the iris being gently raised, the point of a fine pair of scissors was introduced behind the hook, and the raised part snipped off and brought out. There was a slight effusion of blood from the iris, which rendered the eye turbid. The antiphlogistic plan was continued, as above mentioned, and in about a week the effusion was absorbed, and the eye became clear. We then found the pupil in its proper place, and fully the natural size; such as it usually is in a moderate light, and quite circular, except the part near the external angle, towards which it was lengthened a very little, and the margin of that part not so smooth or well defined as the other part of the circumference. He could now discern colours, point out the different objects in a room, and the panes of a window, or the colour of people's clothes at the opposite side of the street. In a few days he went home to the country with a very useful degree of vision. It is now five months since the operation, and I have lately heard from him. He is able to follow his business as usual, and the sight of the eye is as good as before the accident, except that the pupil has not the power of contracting, and it is necessary to wear a shade over it in a strong light."

By this method of operating, the artificial pupil is made with as little mischief to the eye as by most of the other processes, and the opening corresponds with the situation of the natural pupil, which it more nearly resembles than the inconveniently large transverse pupil formed by the operation to which we have just alluded. The lens, too, remaining untouched, the cure must be more rapid, and the restored vision will approach nearer to its pristine condition. If we may may be allowed to pass an opinion on its specific merits, it appears to us to be especially applicable to those instances of obliterated pupil which sometimes occur after the operation of extraction; but that when the pupil has become obliterated by inflammation of the iris, and adhesion of its posterior surface to the capsule of the lens, it cannot be effected in the way proposed by Mr. Moore. Nor, in truth, are we without our suspicion, that even in the case detailed, the lens, by the escape of so large a portion of the vitreous humour actually sunk further back than its usual situation immediately behind the plane of the iris; and that to this circumstance may, perhaps, be ascribed the fact of its having escaped being wounded by the hook or curved scissars. The operation is, however, well worth recording, and adds to our knowledge in the mode of proceeding with this delicate organ.

Art. VII.—*Case of Conversion of the Substance of the Heart, accompanied by the production of a Sac at the Mouth of the Aorta.* By Thomas Spens, M. D. Fellow of the Royal College of Physicians, Edinburgh, and one of the Physicians to the Royal Infirmary.

This appears to have been one of those numerous cases in which active inflammation has gone through all its stages, and the patient has fancied himself well because the immediate urgent symptoms have subsided. The only previous history which could be traced was that, during a severe illness which she had lately gone through, she suffered frequent and long-continued pains in the lower part of the back and belly, extending down the thighs, with a bloody discharge from the rectum and vagina. "The seat of the disease, (continues Dr. Spens) was obscure, but she recovered, and was tolerably well for months." Whether Dr. S. saw her during this illness does not appear. If he did, it is only another proof of the absurdity of attempting to ascertain, as some systematic writers pretend, the exact viscus which is the seat of an inflammatory disease. In violent inflammation in females, nothing is so common as hæmorrhage from the uterus, which seems an attempt at spontaneous cure, but is too often the only symptom attended to, especially if no medical man is consulted. If such was the case in the present instance, we cannot wonder at the quantity of disease found about the cardiac region; all of it, in our opinion, the consequence of previous high inflammation, which so much altered the texture of its connections, as to render it shortly after incapable of performing its functions, and consequently to terminate in death.

Art. VIII.—*Substance of a Clinical Lecture on a Disease of the Valves of the Heart, producing Pulsation of the Jugular Veins.* By GEORGE PEARSON, M.D. F.R.S. In a Letter to Dr. DUNCAN, Professor of the Institutes of Medicine.

Art. IX.—*History of a Disease from Thickened and Cartilaginous Valves of the left Ventricle, and of the Semilunar Valves of the Aorta.* By GEORGE PEARSON, M.D. F.R.S. &c.

THESE two papers do credit to the mode of conducting the clinical studies of St. George's.

Art. X.—*Case of Cynanche Laryngæa;* by JOHN ABERCROMBIE, M.D. Fellow of the Royal College of Surgeons, of Edinburgh.

THIS case is well related, and Dr. Abercrombie succeeded in his treatment of it by early and free bleeding. The subject was a female. Perhaps the greater irritability of that sex may render it more susceptible to remedies.

Such are the contents of this number, the whole of which do credit to the managers and contributors.

---

*A Treatise on the Medicinal Leech; including its Medical and Natural History, with a Description of its Anatomical Structure; also, Remarks upon the Diseases, Preservation and Management of Leeches.* By JAMES RAWLINS JOHNSON, M.D. F.L.S. Member Extraordinary of the Royal Medical Society, Edinburgh. Illustrated with Engravings. 8vo. Longman and Co. London, 1816.

THE Faculty will be much obliged to Dr. Johnson for supplying a desideratum, which, if we may judge by our own feelings, has long existed. Animals so perpetually in our view, and even in use, the scarcity of which has been for some years so severely felt, claim an interest which it is by no means creditable to have so long overlooked. Many of our brethren are excellent naturalists; indeed, there are few of the latter description of philosophers who were not originally educated for medicine, which they have only relinquished on account of the bewitching charms of physics, properly so called. If less attention has of late years been paid to all the more minute structure and habits of many animals, may we not impute it in part to that illustrious naturalist to whom we are indebted for almost every system now in use. The arduous task undertaken by Linnæus of reducing all the objects of nature to classes, orders, genera, species, and varieties, not only excites our astonishment at the boldness of the undertaking, but our gratitude for the manner in which it is executed. But, as no good is unattended with some inconvenience, convenience, so we cannot help thinking that this habit of systematising has made us anxious to undertake too much, and too inattentive to the completion of single objects. Thus we are apt to suppose that it is sufficient to fix on a certain mark for a genus, and the presence of this with some other distinction for a species. Having done thus much, our task seems finished; and the more interesting minutiæ of form and habits are either overlooked or passed over as suited to those only who are contented to confine themselves to a few objects in nature. The motto chosen by our author is a very good lesson against this inattention, and we heartily wish the moderns had followed Aristotle in his designation of animals whom the moderns are in the habit of calling *inferior* or *more imperfect*. In our opinion, *less honoured* or *less attended to*, as used by the Stagerite, is a much more becoming expression for the professors of a religion which teaches that Solomon in all his glory was not arrayed like the lillies of the valley, and that not a sparrow falleth to the ground without the same superintendance as governs the affairs of men.

"Δεῖ μὴ δυσχεραινειν παιδικῶς τὴν περὶ των ἀτιμωτέρων ζῶων ἐπὶσκεψιν; εν πᾶσι γαρ τοῖσ Φυσικοῖς ἐνεστὶ τὶ θαυμαςτὸν.—
"Aristotle: B. i. c. 5."

The first section contains the medical history of the leech. In this we have an account of the period as high as we can trace its introduction into medicine, which Dr. J. supposes cannot be beyond Themison, the founder of the methodic sect who flourished about the beginning of the Christian æra.* From that period, most of the writers by whom the leech is recommended, are named till we approach near enough to the present times. This chapter, like all other collections of historical anecdotes, is more interesting than useful. It, however, makes a necessary part of such a work.

An account of the natural history of the leech follows. We shall not enter into any controversy concerning generic distinctions, as this would rather become a work devoted exclusively to natural history. It is enough to remark that Dr. Johnson excludes the *hirudo complanata* and *stagnalis* from the genus altogether; and, on account of their retractile and tubular tongue, he gives them the appellation *Glossiphonia.* Of these, he makes two species: G. *Tuberculata* (Olim, H. Complanata), and G. *Perata* (Olim H. Stagnalis). His generic character of Hirudo is *Corpus oblongum subrotundum,*

* We are informed, that the lately proposed method of snipping the tail is as old as the time of Pliny.

*anterius et posterius truncatum, muticum, cartilagineum, os caudamque dilatando progrediens.*

"*Hirudines in Rivis, Stagnis, Paludibusque habitantes.*"

The following is the specific character of *Hirudo Medicinalis.*

"Hirudo depressa nigricans, supra lineis flavis sex, intermediis nigro arcuatis, subtus cinerea nigro maculata.

"Oculi decem,* more delineato,

"Long. Pollices tres.

"In Stagnis—Paludibus.

"*Caput*—quiescens subrotundum, progrediens acuminatum.

"*Os*—quoad figuram mutabile, rimam triangularem plerumque exhibens.

"*Cauda*—circularis, complanata, fibris carnosis e puncto centrali divaricatis.

| | |
|---|---|
| "Linnæus, | *Syst. Nat.* XII. 2, p. 1079, n. 2. |
| "———— | *Faun Suec.* 2079. |
| "Hill, | *Hist. Anim.* p. 16. Hirudo nigrescens flavo variegata. |
| "Gesner, | *Pisc.* 425, tab. 425. Hirudo major et varia. |
| "Muller, | *Hist. Vermium*, 2. n. 167, p. 37. |
| "Barbut, | *Genera Vermium*, p. 19, tab. 2, fig. 5. |
| "Weser, | *Amœnitates Academicæ*, tom. vii. p. 42. |
| "Bergmann, | *Act. Stockh.* 1757, p. 308, n. 4, tab. 6, fig. 1, 2. |
| "Gisler, | Ibid. 1758, p. 95. |
| "Saloman, | Ibid. 1760, p. 35. |
| "Shaw, | *Naturalist's Miscellany*, tab. 218. |
| "Pennant, | *British Zoology*, vol. iv. p. 36." |

All the other species are enumerated with the greatest accuracy. We think our author somewhat over-delicate in not giving a different trivial name to H. *sanguisuga.* This is the horse-leech, whose character or mode of feeding gives it a much better claim to H. *vorax*, as is afterwards observed. An English name for each species would also have been desirable, especially, as in describing the habits of some, English terms are introduced. We shall, of course, confine our attention principally to the medicinal leech.

"The medicinal leech is common throughout the whole of Europe, but more so in the southern than in the northern parts. It is about three inches in length; but in the southern parts of America and India it is often found to be six or seven inches. Formerly this species was very abundant in our island; but from

"* No leech has been hitherto described with more than eight eyes. In the H. *medicinalis* and H. *sanguisuga*, no notice has been before taken of an organ of vision."

their their present scarcity, owing to their being more in request among medical men, and to the rapid improvements which have of late years taken place in agriculture, particularly in the draining and cultivation of waste-lands, we are obliged to receive a supply from the continent, chiefly from Bourdeaux and Lisbon.* These leeches differ from the medicinal leech of this country, in being larger, and having the belly of one uniform colour.

"Leeches are observed to take their predominant or ground-colour, from the colour of the soil in which they are found. Mr. Baker, a man of some intelligence, residing in Glastonbury, and who for the last twenty years has been in the habit of collecting large quantities of leeches for sale, informs me that at the Back River, near Glastonbury, they are black, from the peat being of that colour; at Cook's Corner, they are of a reddish cast, from the red peat; and at Auler Moor, where from a deficiency of peat they penetrate the clay, they are yellow. This assertion receives some support from its being remarked of the toad, that if it be much exposed to the light, its colour changes to that of a pale green; but when it remains in the earth, and is not under its influence, it generally acquires the colour of the substance which surrounds it.† The H. *tessulata* is said to vary so much, in respect to colour, that unless it were continually observed, it would be taken for a different species.

"Notwithstanding leeches are thus subject to this change, they still offer spots or lines which are considered permanent, and upon which we may safely rest in giving their specific character.

"The motion of leeches in water resembles that of the eel. They swim in a serpentine direction, and at times with considerable velocity. Besides this, they have a creeping motion, and are capable of moving the anterior extremity of their body, either forward or backward. When the leech wishes to change its place, the circular muscles with which it is furnished are called into action: the diameter of the body being in this way lessened, the extension of the head is effected. Its attachment is then made secure by the sucker terminating that extremity. One point of attachment thus gained, the longitudinal muscles act in their turn, and draw the tail towards the head. The tail is then fixed by means of a similar sucker, and the head, by the re-action of the circular muscles, is again thrown forward. By these alternate movements, the leech is enabled to move on a solid surface with great facility. When the leech quits the bottom of pools, to reach the surface of the water, the body is projected at full length in an oblique position, and is

"* On a moderate calculation, we employ at least one hundred foreign leeches for every British leech. I give this as the opinion of a person who has for many years been a leech-dealer, and whose occupation enables him to give this statement on very satisfactory evidence.

"† Fyfe's Comparative Anatomy, p. 232.

then then moved in a waving direction, upward and downward. By this action, briskly repeated, the leech is thrown forward, and soon accomplishes its purpose.

" In winter, leeches resort to deep water; but in summer they delight in the shallows, where they are more exposed to the influence of the sun. When the weather is very severe in winter, or so dry in summer as to endanger the total drying up of the pools they inhabit, they retire to a considerable depth in the ground, leaving a small aperture to their subterranean habitation. They begin to make their appearance in the water about the latter end of March or the beginning of April. During a bright sunshine they may be seen very actively swimming from place to place; but, should the weather prove cold or cloudy, they confine themselves to the mud. In rainy or windy weather, when the water is agitated, they retire from sight. Just before a thunder-storm, they commonly come up to the surface; and this the leech-gatherers find a good time for collecting them.

" Leeches are said to predict changes in the weather, with so much accuracy as to serve for barometers.

" A clergyman residing in France found that a leech, enclosed in a glass vessel half filled with water, and kept in a window of his chamber, answered every purpose of a barometer. Each morning, he informs us, the leech had shifted its position, in strict unison with the varying state of the atmosphere. From attentive examination, he was enabled to ascertain,—first, that when the weather was about to be serene and pleasant, the leech remained at the bottom of the vessel without the least movement; secondly, that if it was about to rain, in either the fore or afternoon, it mounted to the surface of the water, and there remained until the return of fine weather; thirdly, that on the approach of boisterous weather, it moved in the water with uncommon swiftness, and never ceased from this motion until the wind began to blow; fourthly, that on the approach of weather attended with thunder and rain, it remained out of the water for several days, appearing agitated and restless; fifthly, that it rested constantly at the bottom of the vessel when a frost was about to commence; and sixthly, that during the time of snow or rain, it fixed itself at the neck of the vessel, remaining at perfect rest.* Cowper, the celebrated author of The Task, has asserted that leeches, " in point of the earliest intelligence, are worth all the barometers in the world." Although I cannot agree with our poet, I will not say that leeches are unaffected by the weather, since this is sufficiently proved by their restlessness on its various changes; being affected in like manner with those animals of which the Mantuan bard, in speaking of an approaching shower, gives so natural a description.

" * Vide Supplement to the *Encyclopedie ou Dictionnaire Raisonne des Sciences;* article ' *Sangsue,*' tom. 4, p. 733.

" Aut

"Aut illum surgentem vallibus imis
Aëriæ fugere grues:—aut bucula cœlum
Suspiciens, patulis captavit naribus auras:
Aut arguta lacus circumvolitavit hirundo:
Et veterem in limo ranæ cecinere querelam.
*Georg.* lib. i. v. 374.

"How far the remarks of either the French clergyman or the poet are worthy of notice, will appear by observing vessels in which leeches are contained. Some leeches will be seen in a state of rest, others in motion; some at the bottom of the water, others at the surface. I would therefore ask how it is possible they can furnish any thing like accurate indications of the state of the atmosphere?"

The food of the medicinal leech is said by many writers to consist of worms and larvæ of aquatic insects. This our author shows to be erroneous. It might, indeed, be expected from the uniformity of nature, that the same animal is not fed in such different ways. The error, Dr. J. imputes to confounding this with H. *Sanguisuga.* The following experiments prove, that the H. *medicinalis* and *troctina* confine themselves to blood.

"Having procured a frog, I placed it in a vessel containing half a dozen leeches (H. *medicinalis* and H. *troctina),* in which floated a piece of deal. The poor animal, finding itself surrounded, made every effort, but ineffectually, to reach the upper part of the float; while its enemies pursued it with more than common activity. At length one of the leeches settled on the back, and the others affixed themselves to the legs. On the following morning the frog was found dead, presenting, on different parts of its body, no less than eighteen wounds, all bearing the usual triangular appearance."

Many other experiments were tried, all tending to the same proofs. Larvæ of aquatic insects were also placed in the same vessel, but remained untouched. On the contrary, nothing could exceed the voraciousness of the horse-leech.

"Desirous (says Dr. J.) to ascertain how many of the smaller leeches would be swallowed in a given time, I kept two horse-leeches in separate vessels for a month, supplying them constantly with the *H. vulgaris,* both in its dead and living state.

"During the whole of this period, I must observe, the water was turbid, notwithstanding its occasional renewal, from the vast disengagement of fœcal matter which floated about, having a thread-like appearance.

| *Swallowed by No.* 1. | | *Swallowed by No.* 2. | |
|---|---|---|---|
| June 10, two, dead ...... | 2 | June 10, one living and one dead ...... | 2 |
| 12, two, living ...... | 2 | 11, one, dead ...... | 1 |
| 15, one, living ...... | 1 | 12, one, living ...... | 1 |
| 20, two, dead ...... | 2 | 16, one, living ...... | 1 |
| 21, one, dead ...... | 1 | 17, three dead and one living ...... | 4 |
| 23, one, living ...... | 1 | 19, three, living .... | 3 |
| 25, two, living ...... | 2 | 20, three, living .... | 3 |
| 29, two, living ...... | 2 | July 2, two, dead ...... | 2 |
| July 3, one, living ...... | 1 | 8, two, living ...... | 2 |
| 9, one, living ...... | 1 | 9, one, living ...... | 1 |
| Total, | 15 | Total, | 20 |

"On the fifteenth of June, three *H. sanguisugæ* swallowed three *H. vulgares* On the 20th, I opened the three, and could not, in two, trace the least vestige of a leech. In the third, I found a leech about half digested, surrounded by a fluid, in colour of a deep brown. The intestine in the others was filled with a similar fluid, but much thicker in point of consistence.

"This experiment, with the preceding table, cannot but convince us of the activity of the digestive powers in the *H. sanguisuga.* This indeed may be also determined by a reference to its anatomical structure. In the *H. sanguisuga*, the intestine is more than double the width of that of the *H. medicinalis* and *H. troctina;* and the stomach is not so thickly set with membranous folds or partitions. A difference therefore in regard to food necessarily arises, from this difference of structure.

"To the general law in cold-blooded animals, that digestion proceeds with great slowness,* the experiment just mentioned offers a marked exception."

We cannot forbear transcribing the following, to show how little we can depend on those writers who conceive it their duty to explain every thing in natural history. It is much to be regretted that all authors are not careful to dis-

"* We learn from that indefatigable experimentalist, Spallanzani, that a lizard, after remaining sixteen days in the stomach of a viper, was found to have lost nothing of its original form—a remarkable instance of the extreme slowness of the digestive powers in cold-blooded animals."—This apparent slow digestion is accounted for by Mr. J. not merely from the deficiency of digestive power in the animal under cold, but from the absence of any necessity for food. That great physiologist found that food in the stomach of torpid animals underwent no change during the winter.—Edit.

tinguish what they take on trust from the result of their own experiments.

"Like the hydra of ancient times, leeches are stated to have the remarkable property of re-production. Dr. Shaw observes that this is very conspicuous in the smaller kind of leeches, the *H. stagnalis*, *H. complanata*, and *H. octoculata;* in which animals it almost equals that of the Polype. The doctor's experiments were made in 1773. To use his own words, 'These animals were divided in every possible direction; and the divided parts, after reproduction, were again sub-divided and again re-produced, without the failure of one single instance.'* These experiments I have repeated, but with this wide difference, that I have met with failure in every instance."

The author gives a history of his own experiments, which it is not necessary that we should transcribe. It may not be amiss to add the authority of a celebrated, and, I believe, very faithful, naturalist.

"Many, says Müller, might be easily led into an idea, that an animal whose members, when separated, possess, for a great length of time, their vitality, is capable of re-production; but, he judiciously adds—aliud enim est vitam in singulis partibus aliquamdiu remanere, hasque se movere; aliud in totidem abire animalia: illud pluribus animalium commune est, hoc minus vulgare. In another place this author observes, what is more to the point, that he has endeavoured to ascertain how far this power of re-production in leeches exists; but all his experiments go to prove that leeches have no re-productive power whatever.†"

The interest we have felt in this ingenious little performance has induced us to protract our account longer than such an article might seem to admit. We shall, therefore, conclude with a summary account of what remains.

The leech is said by some naturalists to be infected with its own particular louse; but Dr. J. has not been able to discover any.

Some remarks follow on the mode of propagation, their longevity, vivacious powers, its growth, &c. On these subjects we shall only offer one slight hint to our author, for his further consideration, or our better information.

"The following instances (says he) of the extreme slowness of

---

"* Linn. Trans. v. i. p. 95."

"† 'Plurima (Helminthica) per partitionem naturalem et artificialem transversam propagantur; utramque in *Hydris, Naidibus*, et *Lumbricis*, ego et ante me alii docuere; artificialem in *Hirudinibus* vero, et *Gordiis* frustra tentavi.'—*Hist. Verm.* 1774, p. 9."

its growth, cannot but be admitted as valid proof of its being long-lived, conformably to the general law, that the longer the time an animal takes in arriving to maturity, the greater is the duration of its life."

However general this law may be, we fear, like most others which Lord Bacon attempted to establish in physics, it is liable to many objections. Most birds arrive early at their full growth, yet some of them, even in an unnatural state of confinement, are very long-lived.

The third section is on the anatomical structure of leeches. As this is illustrated with very delicate plates, it would be injustice to offer a description without them. We give great credit to the author for the diligence with which he has detected every part he describes. On the disputed subject of the anus, we are ready to join in the surprise he expresses that the justly celebrated J. Hunter should admit, without more minute examination, that the leech is destitute of that part. This may be a very useful hint to those philosophers whose every word is attended to. Mr. Hunter, before he admitted this as fact, ought to have examined for himself, and it is probable that he saw his error before he died, as we find Sir Everard Home using a different language in his Lectures on the Hunterian Musæum. We cannot, however, help expressing a wish that Dr. Johnson, when speaking of the capacity of leeches to resist cold, had referred to Mr. Hunter's infinitely more philosophical experiments than those which he cites from Bibiena.

We have next a description of the organs of sense, as far as the most diligent research could discover them; and of the mode of breathing, which Dr. J concludes, from very good authority, and his own experiments, is by spiracula.

The internal structure is next minutely traced, and well illustrated by engravings. The hermaphrodite, or rather androgenous character, is well ascertained; and the whole concludes with a chapter on the diseases of this interesting and useful little creature.

To recommend such a performance would be superfluous, as every medical practitioner will find an interest in every part. But, among other uses, we conceive no trifling one to be that, during the dull process of applying leeches, the patient may be entertained with its natural history, and this knowledge being familiar with the younger part of the profession, may instruct as well as induce them to give greater attention to this living charge committed to their care.

*Medico-*

### *Medico-Chirurgical Transactions, published by the Medical and Chirurgical Society of London.* Vol. VI.

*(Continued from vol. 35, p. 418.)*

### *On some Affections of the Larynx, which require the Operation of Bronchotomy.* By William Lawrence, Esq. F.R.S. &c. &c.

We have taken this paper out of its order in the volume, because of its connection with the three concluding ones in our Number for May. The subject may be truly called awful, whether we consider the condition of the patient, or the nature of the proposed operation. After a few preliminary remarks, Mr. L. introduces two cases of his own. At the close of the paper he gives a third, and also a fourth, communicated by Dr. P. Latham.

"In some bodies (says he) which I have examined after death, appearances have been found analogous to those described by the learned physicians just quoted. The patients died of suffocation; but the progress of the complaint was much slower than in those cases,—the symptoms were not acute, nor did the inspection of the parts disclose any evidences of active inflammation. The membrane covering the chordæ vocales was thickened, so as to close the glottis, and a similar thickening extended to a small distance from these parts, accompanied with an œdematous effusion into the cellular substance under the membrane. The epiglottis did not partake of the disorder. In one or two instances, this thickened state of the membrane was the only change of structure observed; but in others, it was attended either with ulceration of the surface near the glottis, appearing as if it had been formed by an abscess which had burst; or with a partial death of one or more of the cartilages of the larynx, viz. the arytenoid, thyroid, or cricoid. The rest of the air passages and the lungs were healthy. Having, within a short time, performed bronchotomy in two cases of the kind just alluded to, I shall shortly relate the particulars of them."

Of these two cases, we shall give the dissection, and some of the author's remarks. The first patient appeared almost in *articulo mortis* when the operation was performed. He was, however, completely relieved in his respiration, and lived eight days, breathing, without the least difficulty, through the wound, from which there was a copious discharge of thick mucus, and afterwards of purulent fluid. On examination after death,—

"The chordæ vocales, sacculi laryngis, epiglottis, &c. were perfectly healthy; their membranous covering, as well as the lining of the trachea, free from every appearance of inflammation; and the rima glottidis of its natural dimensions. There was a small internal internal apperture at the back of the larynx, under the glottis, leading into a cavity on the outside of the membrane, which contained about one half of the cricoid cartilage completely bare and loose. This part had previously undergone the change into bone, which the cartilages of the larynx, at least the thyroid and cricoid, usually experience before the age of the present patient.

"The chest exhibited extensive marks of recent disease, in adhesions of the lungs to the pleuræ, and effusion of whey-coloured fluid with flakes of coagulated lymph. The lungs themselves were also considerably diseased."

The second case was not less formidable, when Mr. Lawrence undertook the operation, which was as soon as he was introduced to the patient. Bronchotomy produced a temporary relief; but the passage was soon plugged up by the viscid secretion, and many circumstances prevented a removal of a portion of the trachea. The following is the account of the examination after death:

"The membrane of the chordæ vocales, sacculi laryngis, and front of the arytenoid cartilages, possessed its natural pale colour, but was thickened and granulated on the surface, so as completely to shut the rima glottidis. The affection, entirely confined to the parts just enumerated, occupied a very inconsiderable extent of the membrane, just enough, indeed, to close the entrance of the trachea. The rest of that tube, the epiglottis, and neighbouring parts, and the contents of the chest and abdomen, were perfectly healthy. A portion of the tube had been cut through longitudinally, at the side of the opening in the trachea, and was nearly detached.

"Considering that no part was found diseased in this woman, except a square inch at most of mucous membrane, I cannot but ascribe her death to obstructed respiration; and think it probable the event would have been different, had more complete relief been afforded by the operation."

This case introduces another from the Edinburgh Journal, which, to render our remarks at the close of the article more perspicuous, we shall call the 3d case. It was more chronic in its history, having existed, to a certain degree, for several months before the patient applied for relief at the Infirmary. The patient (a female) visited some friends half a mile distant, and returned on foot. The symptoms were so much exasperated that she died on the following day, whilst the operation was under contemplation.

Case the 4th is related by Pelletan in the *Clinique Chirurgicale*.

"After an attack of fever, which yielded to the ordinary means, a pain in the throat remained, accompanied with difficulty of swallowing, which increased rapidly, and afterwards with uneasiness in in breathing. Nothing unnatural could be observed on looking into the throat. Blisters were applied externally, but the disorder gained ground; and the progress towards suffocation becoming accelerated, Pelletan opened the trachea. It was too late, for the woman died the same day. The membrane of the epiglottis was so much swollen, as to give that organ a globular figure. The membranous lining of the larynx and pharynx was equally swollen, and increased in density. The opening of the glottis was reduced to less than one third of its natural size."

Many other instances are produced, some of which were under Mr. Lawrence's inspection after death; but, as he did not witness the early symptoms, we shall not produce them: nor shall we notice his learned references. Though all of them might not be necessary, they are interesting in showing the state of surgery, and the opinions of practitioners at different periods of the world. We only transcribe the following, because we conceive it to be not less valuable for point than authenticity. It is extracted from the 416th number of Phil. Trans.

"I was called (says Dr. Martin) to a young lad, who, being in such a good state of health as to be making a visit to some of his comrades in another street, was all of a sudden taken ill of a violent trouble in his throat, in which, however, I could see nothing wrong; the amygdalæ and other parts in view being in all appearance sound enough, but only looking a little drier than ordinary, without any external tumour appearing about the larynx; and no considerable frequency or strength in his pulse. But he had great pain and a dyspnœa, with an impossibility of swallowing either solids or liquids, every thing returning forcibly by the mouth and nose, when he made an effort to get it over. From all which I reckoned it an angina of one of the worst kinds, *sine apparente tumore* (see Hippocr. Prognost. 23, 3, and Prænot. Coac. 3, 96.) and the seat of the disease in the larynx, and the fibres common to it and the top of the gullet.

"Notwithstanding repeated bloodings, blistering betwixt the shoulders, cupping, &c. whereof it is needless to give you a particular detail, the disease continued so obstinate, and the patient so like to suffocate, that next day, in the afternoon, his friends, though very averse in the morning, when I first proposed the piercing the windpipe, at length earnestly desired that the operation might be performed; and the poor lad bade us to try any experiment to preserve his life. He had good reason to do so, for, indeed, in all probability, in a few hours, he would have been strangled to death most miserably, *constante mente integrisque sensibus*, as the elegant Fernelius expresseth it, (Patholog. v. 9.) whence you see it was not out of an itching desire of making experiments, or a wanton officiousness, that we directly set about the operation, which was done with such success that in less than

 four four days, his breathing being perfectly easy, and his deglutition almost so, we removed the canula, and left the glottis to do its own office.

"The patient was soon perfectly recovered: he breathes, speaks, eats, drinks, and performs all the other offices of life, and goes about his calling as formerly. And now I cannot but notice the needless pain some writers are in about healing up the wound by bandaging, stitching, &c. for we found it easily to fill up of itself in very few days, by only dressing it every other day or so with a soft tent made less and less every dressing, and armed, in the common way, with liniment. arcæi."

In the subsequent case, related by Mr. Lawrence, he was completely successful; nor can there be a doubt that to *his* skill and courage the patient is indebted for the preservation of life.

After a few days' hoarseness, "on the 7th of July he began to experience a difficulty of breathing, and, to use his wife's expression, *to hoop;* that is, to draw his breath with a peculiar sound. The voice was still more affected, and reduced to a kind of whisper. He was bled and purged on the 8th. At one in the morning of Wednesday the 12th, he again became much worse; the difficulty of fetching his breath was so great, that his wife said he was like a person running mad, not remaining quiet for a moment, but walking and moving about incessantly. Yet he particularly observed that this was his only grievance; and that if the stoppage in his throat could be removed, he should be well. He took an emetic, of which the operation rather relieved him; a very large blister was applied to the chest, and the whole throat was covered by another. The difficulty of breathing still increasing, in spite of the full action of these blisters, and the danger of suffocation being extremely urgent, he was sent by Mr. Parkinson, of Hoxton, to St. Bartholomew's Hospital, where I saw him at six in the evening. The distress of breathing was extreme; every inspiration performed with great effort, and the assistance of all the auxiliary powers was attended with a loud hooping noise, audible across the hospital square. He sate up in bed, shifting about incessantly to get breath, and agitated by the momentary expectation of suffocation; the occurrence of which, without some immediate relief, seemed close at hand. Sweat poured down in streams from the whole body: the pulse was 120, full, strong, and intermittent. He had no difficulty nor pain in swallowing, and felt no inconvenience from fully distending the chest. There was a little coughing occasionally, excited by a colourless mucus about the larynx. Judging, from the circumstances just detailed, that the affection was confined to the opening of the larynx, and that the source of the patient's danger was a mechanical impediment to respiration, which bleeding and other evacuations, although fully justified by the state of the pulse, could not be expected to remove, I immediately diately determined on bronchotomy, receiving, in this determination, the sanction of my friends, Mr. Wheeler, the apothecary to the hospital, and Mr. Langstaff, who kindly favoured me with their assistance. I made a perpendicular incision, cut through the cricoid cartilage, and neighbouring part of the trachea, and removed a sufficient portion of these parts to leave a free opening for respiration. The blistered state of the skin, the depth of the parts in a short and thick neck, the rapid motions of the larynx, and the entrance of blood into the tube from vessels divided in exposing it, produced greater difficulties in the operation than a person would expect, who formed his opinion from the ease with which it is accomplished in the dead subject. Two small arteries bled freely: one of them was tied; but the other could not be secured, on account of its lying completely under the edge of the cricöid cartilage: it was therefore left, the patient bending forwards that the blood might not flow into the trachea. He breathed quite easily through the artificial opening; all the agitation and distress ceased; the skin became cool, and the pulse softer. Soon after he had some sleep, but did not rest much during the night. He took a saline mixture, with small doses of the tartrite of anatomy. The pulse was rapid, and intermittent for two or three days, but he was free from fever. Breathing was performed entirely through the wound, and the voice, consequently, was completely lost. There was a copious mucous and purulent discharge from the trachea and wound. On the 21st he was sufficiently recovered to get up. By holding the edges of the wound together, he could breathe through the larynx and speak, but there was still a feeling of difficulty, which made it necessary to open the wound again in a short time. The 5th of August was the last day on which any air came through the wound, which had completely cicatrized on the 10th, when he was discharged from the hospital perfectly recovered, excepting that the voice still remained rough and hoarse."

Dr. P. Latham's case concludes the paper. Of this we shall not think it necessary to give any epitome or extract, not because the case proved fatal, but because the writer remarks that "the patient and her friends were of the low Irish, and each gave a different answer to the questions (concerning the previous symptoms) proposed to them.

Having given this very copious account of a most important paper, we shall transcribe the judicious remarks of the author.

"The facts (says he) which have been related and alluded to in this paper, seem to me to justify the following conclusions, viz.

"1. That the larynx is subject to affections differing considerably in the nature of their symptoms, and in their progress; but resembling each other in their ultimate effect, of obstructing the passage by which air is received into the chest.

"2. That the difficulty of breathing amounting to a sense of suffocation, suffocation, the sound produced by the passage of the air, the affection of the voice, which is either extremely hoarse or reduced to a scarcely audible whisper, in many cases pain of the throat, and difficulty of swallowing, together with the absence of symptoms indicating affection of any other organs, are the signs by which this obstruction may be recognised.

"3. That the impeded state of respiration causes a violent constitutional disturbance in the acute cynanche laryngea, while it has a general debilitating influence in the more chronic forms of the disorder; and that these effects are in themselves fatal, after a certain time, even if the original obstruction be obviated.

"4. That local and general bleeding, blisters, and the various internal means, are usually inefficacious.

"5. That the operation of bronchotomy, by providing an artificial opening for the air, produces complete relief, but, for the reasons mentioned under the third head, it is ineffectual, unless performed very early.

"6. That the operation is free from danger, has been many times successfully performed, and has not in any instance produced unpleasant consequences."

Some useful observations follow on the mode of conducting the operation.

In a review of the above remarks, it is impossible to object to the main proposition of an early resort to the operation. But, in another part of the paper, the author proposes that it should precede the usual remedies of bleeding, purging, and blistering. On this we offer at least a few hints.

The main question to be considered is, Whether the disease is purely inflammatory or not? That it is in some cases cannot be questioned, because Dr. Roberts cured his patient by early and free blood-letting (see page 413 of our last Number); and we have shown that this practice has been sanctioned by the ablest physicians in all ages. If it has been found insufficient, the operation is not only justifiable but imperiously demanded. But may not cases occur in which the operation should precede every attempt? To this we answer, that in hospital cases we think they often may, because there, it frequently happens that the case may not be seen till it has assumed a chronic form; that is, till the more urgent symptoms of inflammation have subsided, and the only remaining danger is suffocation from an affection of the larynx, which obstructs the passage of air into the chest, or from such a quantity of secretion as must interrupt the ordinary means of respiration. But in the history of cases in private life, accurately described, the marks of inflammation, *ab initio*, have usually been sufficient

 to to authorise the trial of every antiphlogistic means, and even to demand them, before an operation should be attempted on a part, the irritability of which will be greatly increased by the neighbouring inflammation.

On the third proposition we could wish Mr. Lawrence had dwelt longer. We have seen that in acute cynanche the influence on the constitution is sometimes so violent as to induce death, even when the patient has recovered respiration. There is, therefore, reason to believe that in such cases death has been induced by the high action which has been excited, without ending in any of the common effects produced by such cause; that is, without terminating in either adhesion, suppuration, or effusion; for the two patients mentioned by Dr. Baillie breathed freely before death, yet one of them, on a former occasion, had been relieved by free bleeding in the early stage of the disease.

In what may be called the more advanced stage of the disease, the first inquiry should be, whether, in the beginning, high inflammation existed. In all the cases related in this paper, as examined after death, more marks of the sequelæ of inflammation were found than are necessary to ascertain its existence. In the first, there was purulent fluid during life; and the evidence of abscess at the back of the larynx discovered after death. In the second, the same and neighbouring parts, though pale, were thickened with a granulated surface. These are sufficient, in our opinion, to prove a previous violent inflammation, which had probably subsided before the patient arrived at the hospital; and we have no history of the preceding symptoms. In the third case, from the Edinburgh Medical Journal, dissection proved evident marks of early inflammation. It was probably less violent than in some other cases, and, as it terminated in suppuration, the patient was enabled to protract a wretched existence a few months longer. This case was no doubt similar to those mentioned by the learned president in his note transcribed p. 415 of our last volume. If the history of the disease does not furnish the symptoms of high inflammation in that part, we must remark again the difficulty of gaining accurate histories from the recollection of the complaints of a servant. The dissection shows phenomena which nothing but inflammation can produce. Pelletan's case, though described as chronic, and though the writer of the paper before us, considers it as a slow affection, commenced with an attack of fever. At this time, probably, high inflammation was excited about the epiglottis and larynx, the parts were thickened, and that thickening not subsiding

 sufficiently sufficiently for the necessary action of the parts, induced all the chronic symptoms.

The next case, from the Philosophical Transactions, is peculiarly interesting, not only on account of the success of the practice, but because the author shows how much he was assisted by a reference to those writers who are at present too much undervalued, or too little attended to. Though the writer has not mentioned Celsus, probably because he considered him as a copyist of the Greek physicians, yet it is impossible not to mark the similarity of their observations. All the parts in view, says Dr. Martin, appeared healthy, only a little drier than ordinary. Celsus's words are, "Interdum neque tumor nec rubor ullus apparet sed corpus aridum est, vix spiritus trahitur, membra solvuntur." This he considers the most dangerous form of the disease.

We return now to Mr. Lawrence's successful case. The first symptoms were on the 7th of February, and so considerable, that the patient in breathing was said to hoop, and the voice was reduced to a whisper. On the following day he was bled and purged. The symptoms were somewhat relieved; but on the fourth day afterwards, at one in the morning (the usual period for paroxysms, whether in the beginning or return of acute diseases), the difficulty of breathing was so great that Mr. Parkinson sent his patient to the hospital, and the same evening the operation was performed. There was a copious mucous and purulent discharge from the trachea and wound. When the patient recovered, his voice remained hoarse and rough. Should we not, from all these symptoms, say that the disease was, *ab initio*, high inflammation; but, either from the constitution or habits of the patient, or from the early bleeding and purging, that inflammation was sufficiently reduced to terminate in an increased and altered secretion. Probably some loss of substance, or at least some alteration of structure, had produced the hoarseness, which remained after all the other symptoms had subsided.

On the whole, we cannot help believing that the proximate cause of all the symptoms in this disease is high inflammation, unrelieved by continuous sympathy, and, for the most part, unaltered by the usual termination of effusion, adhesion, or suppuration. That in this its worst form it kills by exciting higher action than the system can support, in which case the patient dies from high irritation, as we see after some operations performed in high health. That, if the termination is in adhesion, it produces croup; if in effusion, strangulation; if in suppuration, either an abscess is formed, formed, or the surface ulcerates, or the common mucous secretion is increased, and becomes purulent,—in all which cases the disease becomes more or less chronic. That, if the disease has lately become more frequent, the same may be said of all other inflammatory complaints. That the proper remedy is very early and very free bleeding, which, in our opinion, should precede bronchotomy, or, if the case is very urgent, that both should be performed at the same time. Lastly, that, though fatal cases may occur, even under all these and every other mode of treatment, yet the same may be said of many other inflammatory diseases, which sometimes prove fatal under the best management hitherto discovered.

*Case of Obstruction in the large Intestines, occasioned by a Biliary Calculus of extraordinary Size.* By H. L. Thomas, Esq. F.R.S.

This disease was suspected to be a strangulated umbilical hernia; and the interruption to the action of the bowels, with violent pain, having continued to the fifth day, Mr. Thomas's chirurgical aid was required.

"The hernia (says Mr. Thomas) presented a tumour of a pyramidal shape, somewhat flattened at the apex, and of the bulk of a moderate-sized lemon: it was not so painful to the touch as the other parts of the abdominal parietes, and, from the tenuity of the integuments, the contents were readily ascertained to consist of both omentum and intestine, in many parts firmly adherent to the inner surface of the sac. The hernia had never been reduced since its first formation, which took place, suddenly, fourteen years before, upon a violent effort in attempting to raise a heavy weight from the ground. There had been no perceptible increase in the size of the tumour till within the last two years, when the habit generally had become more full and corpulent in consequence of her taking less bodily exercise than what she had formerly been accustomed to."

It appears that the hernia was reduced, but still the uneasiness continued till a copious evacuation took place.

"Upon the removal of the soiled linen from the bed, a hard substance, of a considerable size, was discovered in the fæces, which, upon a superficial examination, appeared to possess the properties common to biliary concretions. This notion was fully confirmed by the subjoined analysis, by Dr. Marcet, who, with his accustomed alacrity and zeal in the pursuit of science, readily undertook the task of ascertaining its component parts.

"'It was of an oval shape, nearly regular, smooth at its surface, and of a whitish or pale yellowish colour: it weighed 228 grains, and was specifically lighter than water, as appeared from the calculus floating upon the surface of that fluid. In its longer longer diameter it measured 1·6 inch, and in its smaller diameter 1·1 inch, the circumference in that direction being exactly 3 3 inch.

" 'On being cut through, with a view to examine its internal structure, it appeared to consist of concentric layers or successive depositions of calculous matter, of various thickness; small quantities of a dark brown spongy friable substance being interposed between the strata. In their fracture the strata exhibited an appearance of radiating laminæ, like spermaceti; and the brown matter was strewed here and there, with crystalline particles of a spermaceti-like substance. The calculus was altogether fusible by heat and combustible: when ignited, it burnt with great vividness, leaving minute portions of an alkaline ash. When digested with alcohol, it readily dissolved, with the exception of a little brownish matter, forming a transparent solution, which, on cooling, congealed, and exhibited a congeries of crystalline transparent laminæ, which dissolved again on applying heat. A portion of the calculus being boiled in a solution of caustic potash, imparted to it a greenish colour; yet but a very minute portion of the calculous matter was dissolved in the alkaline menstruum, and on adding muriatic or nitric acid to this solution, no precipitation whatever took place. The brown interposed substance, or colouring matter of the calculus, formed but a very small proportion of the mass, and consisted probably of vitiated bile. It conveyed at first the idea of coagulated blood, but, I believe, without foundation.' "

This case is certainly interesting; but, from the analysis of this substance, the term *calculus* seems very inconsistent, and not at all applicable to a formation of this kind. A compound "altogether fusible by heat," and so far totally combustible as to burn away, leaving nothing behind it but "minute portions of an *alkaline* ash," can scarcely be ranked among calculi of any description whatever. We might, with equal propriety, give the same appellation to another animal concretion, the spermaceti from the cranium of the physeter macrocephalus, to which this biliary accumulation seems to have a very near analogy. Dr. Marcet does not hint at the nature of the alkaline ash, nor mention whether any part of it was soluble; nor does he state the melting point of the concretion itself. Each species of animal fat possesses its own specific property of melting at a given degree of heat: thus, hog's fat melts at 97°, spermaceti at 112°; and hence it may be inferred, that such an extraordinary production as this unctuous and *inflammable* stone will be found to resemble what is termed adipocire, in other respects, besides its point of liquefaction.

It would seem as if mere stagnation of animal matter in any part of the intestines were sufficient to form adipocere in the manner Sir Everard Home has traced in the cœcum.

MEDICAL

